# Brazilian Protocol for Sexually Transmitted Infections, 2020: infections that cause genital ulcers

**DOI:** 10.1590/0037-8682-663-2020

**Published:** 2021-05-17

**Authors:** Mauro Cunha Ramos, José Carlos Sardinha, Herculano Duarte Ramos de Alencar, Mayra Gonçalves Aragón, Leonor Henriette de Lannoy

**Affiliations:** 1 Clínica Privada, Pesquisador Autônomo, Porto Alegre, RS, Brasil.; 2 Fundação de Dermatologia Tropical e Venerologia Alfredo da Matta, Manaus, AM, Brasil.; 3 Secretaria de Estado de Saúde de São Paulo, Programa Estadual de DST/Aids, São Paulo, SP, Brasil.; 4 Ministério da Saúde, Secretaria de Vigilância em Saúde, Brasília, DF, Brasil.; 5 Universidade Federal do Espírito Santo, Programa de Pós-Graduação em Doenças Infecciosas, Vitória, ES, Brasil.

**Keywords:** Syphilis, Chancre, Genital herpes, Chancroid, Lymphogranuloma venereum, Granuloma inguinale

## Abstract

Infections that cause genital ulcers are one of the themes comprising the Clinical Protocol and Therapeutic Guidelines for Comprehensive Care for People with Sexually Transmitted Infections, published by the Brazilian Ministry of Health in 2020. The Protocol and Guidelines have been developed based on scientific evidence and validated in discussions with specialists. This article addresses clinical genital ulcer syndrome caused by sexually transmitted infections and its most common etiological agents: *Treponema pallidum* (syphilis), herpes simplex virus-2 (genital herpes) and herpes simplex virus-1 (perioral herpes), *Haemophilus ducreyi* (chancroid), *Chlamydia trachomatis* serotypes L1, L2 and L3 (lymphogranuloma venereum), and *Klebsiella granulomatis* (donovanosis). Epidemiological and clinical aspects of these infections and guidelines for their diagnosis and treatment are presented, including strategies for surveillance, prevention, and control actions to support health managers and professionals in the qualification of care.

## Highlighted excerpt

Approximately 70% of the genital ulcers attended in specialized clinics are due to sexually transmitted infections, particularly in adolescents and young adults.

## FOREWORD

This article addresses the infections that cause genital ulcers, a subject that comprises the Clinical Protocol and Therapeutic Guidelines (PCDT) for Comprehensive Care for People with Sexually Transmitted Infections (STI), published by the Health Surveillance Secretariat of the Brazilian Ministry of Health in 2020^1^. To draft this document, a selection of available evidence was analyzed and discussed by a panel of specialists. The National Committee for Health Technology Incorporation in the Brazilian National Health System (Conitec) approved this document^2^. 

## EPIDEMIOLOGICAL ASPECTS

Approximately 70% of the genital ulcers attended in specialized clinics are due to STI, particularly in adolescents and young adults^3^. As a syndrome, genital ulcers are not a compulsory reporting condition in Brazil, making it difficult to quantify their occurrence. They are, however, a frequent cause of consultations and are a significant cofactor for human immunodeficiency virus (HIV) transmission^4^. Genital ulcers can have different causes, such as other infections, trauma, inflammatory diseases (e.g., pemphigus, erythema multiforme, contact dermatitis, erosive lichen planus, or fixed drug eruption), and neoplastic lesions (e.g., squamous cell carcinoma or other neoplasms). The latter is particularly relevant in older adults and those with immunodepression^1^. 

Establishing the etiologic agent on a clinical basis is difficult due to polymorphism in presentations^5^. The availability of accurate and rapid diagnostic tests varies according to different agents and different health systems scenarios. For this reason, the World Health Organization has recommended the syndromic treatment, adopted by several countries^3^ ^,^ ^6^ ^,^ ^7^. 

The most common agent found in genital ulcers is the herpes simplex virus (HSV), type 1 (HSV-1) and type 2 (HSV-2). They are DNA viruses that belong to the Herpesviridae family^8^. The second most common agent is *Treponema pallidum*, which causes syphilis. These agents can also be found in association^3^ ^,^ ^9^. Brazil, similarly to other countries^10^, faces a significant increase in syphilis cases since 2017. In 2018, the government reported a detection rate of acquired syphilis of 75.8 cases per 100,000 inhabitants^10^. 

Chancroid is caused by *Haemophilus ducreyi*, a sexually transmitted Gram-negative bacterium. The condition, which occurs most frequently in men and in tropical regions^1^ ^,^ ^11^, has been significantly diminishing, possibly due to the syndromic approach introduction^12^. A systematic review carried out with publications from 1980 to 2014 revealed that after the year 2000, up to 15% of genital ulcers were chancroids, with 14 studies conducted in 13 countries^6^. Since 2000, chancroid cases have been sporadic in Europe^11^. In a Brazilian study at a specialized STI center in Manaus, no chancroid case was identified in 434 genital ulcers analyzed consecutively without prior selection^7^. 

Lymphogranuloma venereum is caused by *Chlamydia trachomatis*, the highly invasive serotypes L1, L2, and L3. Transmission is also attributed to asymptomatic people^13^ ^,^ ^14^. It is an endemic condition among men who have sex with men (MSM) in Europe, predominantly anogenital and rectal infections^14^. Lymphogranuloma venereum outbreaks in high-income countries and MSM (mainly by the L2b variant) have been associated with unprotected receptive anal exposure and HIV infection^15^. Sexual activity facilitated by global travel, online social networking, and pre-exposure prophylaxis of HIV infection is believed to have contributed to its reemergence^15^. 

Donovanosis is caused by *Klebsiella granulomatis*, a sporadic condition most commonly found in countries with tropical and subtropical climates. Associated with sexual transmission, it has low transmissibility, and its transmission mechanisms are not well known^16^.

## CLINICAL ASPECTS

The clinical aspects of genital ulcers are very varied and have low sensitivity and specificity regarding the etiological agent^9^. The diagnosis based on clinical impression showed positive predictive values of 30.9% for syphilis and 32.7% for chancroid, with no clinical correlation for cases with mixed chancre in Brazil^9^. Even so, the characteristics of genital ulcers should be evaluated and vary according to the etiological agent.

### Herpes simplex virus 1 and 2

Infection caused by HSV-1 and HSV-2 sometimes presents a symptomatic primoinfection, which occurs with incubation of six days on average and a duration of approximately 20 days^8^ ^,^ ^17^ ^,^ ^18^. It is characterized by numerous vesicles that evolve into painful, ulcerated lesions, whose bottoms are covered with a yellowish coating. These lesions are accompanied by general malaise, fever, myalgia, painful regional enlarged lymph nodes, and severe urinary symptoms, especially in women^18^. 

Once the infection occurs, the virus is transported along the peripheral nerves' axons to sensitive ganglia. It enters latency, a state in which it can persist for life or suffer reactivations. Reactivated, the virus migrates to the mucous and cutaneous surfaces, again through the sensory nerves, and can be eliminated asymptomatically or cause episodes of recurrent lesions. The outbreaks, which may be the first manifestation, are spontaneous or induced by several factors (for example, exposure to ultraviolet radiation, infections, use of drugs, immunodeficiency, or physical or emotional stress). Recurrent manifestations are more frequent by HSV-2 than HSV-1^17^. Most infected individuals will experience a recurrence in less than one year, and with each subsequent year, their intensity and average number decrease by about one event per year. These recurrences are lighter and last less. They are often preceded by prodromal symptoms such as itching, paresthesia, or pain at the site of lesions near the primoinfection area. Initially, they consist of vesicles of citrine content grouped in clusters on an erythematous base. The vesicles are rarely found in the mucous membranes because they rupture more quickly and originate polycyclic ulcers that regress spontaneously in approximately seven to ten days^17^ ^,^ ^18^. 

### Syphilis

Chronic affection transmitted by sexual contact, whether genital, anal, or oral, or even by vertical transmission^1^ ^,^ ^19^ ^,^ ^20^. It alternates periods of latency and clinical manifestations, often cutaneo-mucous lesions, and can affect any organ with transitory or definitive manifestations, depending on the organ affected^13^. The classic genital ulcer, called hard chancre, typically occurs as a single, painless ulcer with a clean bottom and an infiltrated base at the entrance site of *T. pallidum* ^9^ ^,^ ^20^. More common in genital organs, the ulcer can go unnoticed, especially when located in cavities such as the interior of the vagina, perianal and rectal regions, and oral cavity. The incubation period is from ten to 90 days, with an average of three weeks. The hard chancre disappears without scarring, with or without treatment, in approximately three to eight weeks. Increased regional lymph nodes typically accompany it, often unilateral, usually multiple, with one standing out for its larger size. They are painless, of elastic consistency, and nonsuppurative^19^ ^,^ ^20^ ^,^ ^21^.

### Chancroid

Very painful genital or perianal ulcers typically characterize soft chancre or Ducrey's chancre. It rarely occurs in the oral cavity or other regions of the tegument. It has an irregular border and unclean bottom, covered with a yellowish, necrotic, and fetid coating. When removed, it reveals friable granulation tissue and a non-infiltrated base. The most frequent locations in men are the frenulum and the balanoprepucial groove, and in women, the furcula and the inner face of the inner and outer lips^12^. Satellite lesions by autoinoculation are common, as well as disfiguring scars. It is accompanied by inguinal adenitis in 30% to 50% of cases, a unilateral inflammatory bubo that tends to fistulize through a single orifice. Tense and fluctuating lymph nodes can be relieved by needle aspiration. The drainage or excision of the affected lymph nodes is contraindicated. They may attack deep lymph node chains in the form of systemic disease^11^. 

### Lymphogranuloma venereum

It presents ulcers that usually go unnoticed by the infected person or the health professional. Its evolution occurs in three phases: inoculation, regional lymphatic dissemination, and sequelae.

The inoculation phase starts with a papule, a pustule, or a small painless ulcer, which disappears without leaving sequelae. It may occur in the rectum and rarely in the urethra or cervix. Depending on its location, it may present mucopurulent exudate^14^ ^,^ ^22^. The regional lymphatic dissemination phase develops from one to six weeks after infection. Especially in women, the affected ganglia chain depends on the location of the initial lesion. In men, inguinal lymphadenopathy represents the main reason for consultation and is unilateral in 70% of cases^14^ ^,^ ^22^. In the last clinical phase of the condition, called the sequelae phase, ganglia's involvement evolves with a fusion of lymph nodes into a large mass, with liquefaction and fistulation by multiple holes. Orogenital contact can cause ulcerative glossitis with regional lymphadenopathy. It may be accompanied by proctitis or proctocolitis, sometimes simulating rectal cancer. The illness may be accompanied by general symptoms such as fever, malaise, anorexia, weight loss, arthralgia, night sweats, and meningism. In case of initial lesions in the cervix or rectovaginal pouch, lymphatic drainage is performed by pararectal ganglia, with permanent damage to the lymphatic network. The chronic lymphatic obstruction leads to genital elephantiasis, which in women is called esthiomene. Rectal, vaginal, or bladder fistulas may occur, as well as orificial stenosis, events that should always suggest the diagnosis of lymphogranuloma venereum^14^ ^,^ ^22^. 

### Donovanosis

The original lesion may vary in appearance. It can start with a flat edge ulcer that evolves to hypertrophic or vegetating ulcer, with granular bottom, single or multiple, well delimited, that grows slowly and progressively. It has a bright red aspect and easy bleeding to manipulation. There is a predilection for skin or mucous folds, with frequent "mirror configuration"^23^. Adenitis does not occur, but pseudobuboes may appear in the inguinal region (subcutaneous granulomatous nodules). Genital elephantiasis is a late sequel by obstructive lymphatic phenomena. The extragenital spread is rare and most often occurs from primary genital or perigenital lesions^16^ ^,^ ^23^. The differential diagnosis of donovanosis includes syphilis, chancroid, tuberculosis, and other granulomatous diseases, cutaneous amebiasis, ulcerated neoplasms, and American tegumentary leishmaniasis^1^ ^,^ ^16^. 

## DIAGNOSIS

The assistance to people with lesions presents particularities. The interview must be conducted in an environment of privacy, in an empathetic manner, and without value judgments, either through speech or non-verbal language. Inquiries about sexual practices and identification of STI risk factors, such as age under 30, history of new or multiple sexual partnerships (especially in the past three months), sexual partnerships with STI, prior or concurrent STI, and irregular condom use are required^9^ ^,^ ^24^ ^,^ ^25^. Psychoactive drugs, anonymous or group sexual encounters, professional sexual activity, sexual abuse, street situation, or liberty deprivation must be considered. Populations with difficult access to services, such as transexuals, deserve special attention.

The etiological diagnosis of genital ulcers based on anamnesis and physical examination can be imprecise; therefore, it is important to use diagnostic tests whenever they are available^9^ ^,^ ^26^. These tests only have value if the results are immediately available because treatment institution should not be postponed depending on the results. These tests' sensitivity is variable due to technical particularities and relies on the number of pathogens in the sample obtained^9^ ^,^ ^27^.

### Herpes simplex virus HSV-1 and HSV-2

Serological tests for HSV-1 and HSV-2 detections are available. Although they are of little help in diagnosis, identifying the viral type can help evaluate prognosis and counseling. It is estimated that HSV-2 has greater transmissibility, including transmission from the pregnant woman to the newborn^9^ ^,^ ^24^.

 Viral culture, reserved for research environments and considered a gold standard exam, is being replaced by tests based on nucleic acid amplification test (NAAT), which have greater sensitivity and practicality^26^.

### Treponema pallidum

Dark-field microscopy can allow the identification of spirochetes with characteristic shapes and movements. Lymph obtained by the expression is used, ideally without bleeding, which must be analyzed immediately. The use of material obtained from lesions of the oral cavity is inappropriate due to the presence of saprophytic spirochaetes unrelated to syphilis. Direct immunofluorescence or special stains require longer processing and will hardly be useful in clinical context. Identification of genital lesions indicates serological investigation for diagnostic clarification and screening of concomitant STI. There are two types of serological tests, treponemal and nontreponemal. Among the treponemal tests, the following can be mentioned: rapid tests by immunochromatography, treponemal fluorescent antibody-absorption (FTA-Abs), *T. pallidum* particle agglutination assay (TPHA), electrochemiluminescence, and the enzyme-linked immunosorbent assay (ELISA). Nontreponemal tests, such as the venereal disease research laboratory (VDRL) and rapid plasma reagin (RPR), have particular importance in the serological follow-up^19^ ^,^ ^20^ ^,^ ^26^. Treponemal tests become positive, on average, from one to three weeks from lesion onset, slightly earlier than nontreponemal tests. The rapid tests, distributed by the Ministry of Health, are practical, safe, and easy to perform by trained professionals. They provide results in up to 30 minutes and allow diagnostic support, decision making at the time of consultation, and immediate treatment^27^.

### Haemophilus ducreyi

Smear microscopy of lesions with abundant exudate may allow identification of organized streptobacilli two by two, a configuration compared to "train tracks". This examination has much lower sensitivity and specificity than culture, a procedure with many demands and challenging performance. DNA amplification techniques, such as NAAT, are now considered the gold standard for detecting *H. ducreyi*, with sensitivity up to 98.4%. Some kits for polymerase chain reaction (PCR) have the advantage of simultaneously testing other agents, such as *T. pallidum* and HSV^9^ ^,^ ^11^ ^,^ ^12^ ^,^ ^26^.

### Chlamydia trachomatis

NAAT tests, which include PCR and ligase chain reaction, are the recommended methods for detecting *C. trachomatis* in material collected from ulcer specimens, rectum, bubo aspirates, pharynx, biopsy specimens, and urine samples, which are little available in the routine.^14^ ^,^ ^21^ ^,^ ^28^ Serological tests are of little importance for the identification of *C. trachomatis* ^9^ ^,^ ^26^ *.*

### Klebsiella granulomatis

The diagnosis is established by identifying Donovan bodies in the microscopy of tissue fragment smears or their anatomopathological study, stained by Giemsa, Leishman's, Wright's, or Rapi-Diff methods. It is a microorganism of difficult culture, and NAATs are available, especially in research institutions^9^ ^,^ ^16^ ^,^ ^26^ ^,^ ^29^.

## TREATMENT

STIs are the main cause of genital ulcers and should invariably compose the diagnostic hypotheses, especially in the presence of epidemiological elements. Longer developing ulcers of more than four weeks in duration require a more careful evaluation since they may be related to neoplastic processes, chronic infectious diseases, or immunosuppression. Most of the time, these processes require specialized care, biopsy, and histopathological studies.

STIs' importance as a public health issue lies in their high frequency of occurrence and the need to interrupt transmission. The use of laboratory tests, although desirable, has limitations. In genital ulcers with STI suspicion, it is not acceptable to delay the treatment depending on laboratory test results. The use of combinations of therapeutic options to treat one or more agents is desirable in some scenarios^9^ ^,^ ^19^ ^,^ ^20^ ^,^ ^24^ ^,^ ^25^ ^,^ ^30^. 

**FIGURE 1:**
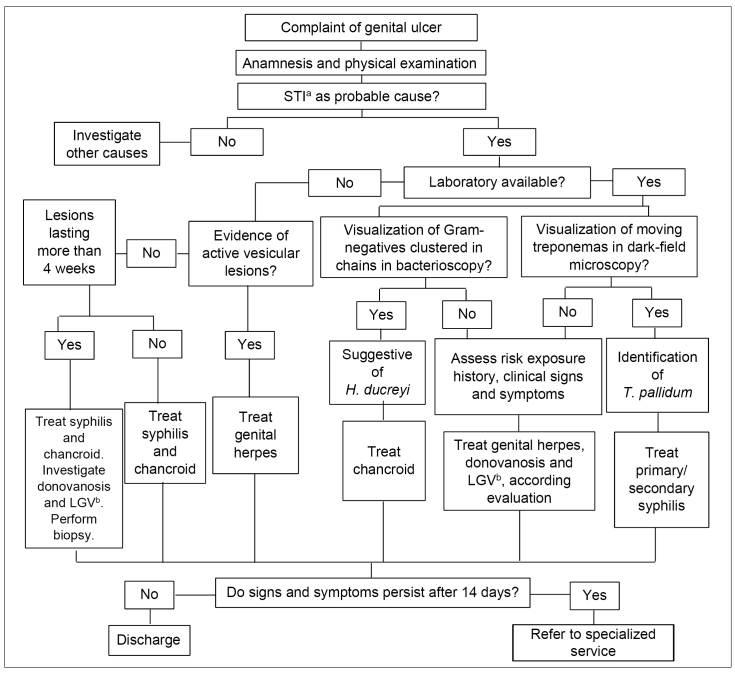
Recommendations for the management of genital ulcer infections.

**Source:** adapted from the Clinical Protocol and Therapeutic Guidelines for Comprehensive Care for People with Sexually Transmitted Infections, 2020^1^.

**Notes:** a) STI: sexually transmitted infections; b) LGV: lymphogranuloma venereum.

Figure 1 presents guidelines for the management of infections that cause genital ulcers^1^. Due to the significant reduction in the incidence of chancroid^7^, the lack of epidemiological surveillance of this condition^31^ and the constant need to update the recommendations, the Ministry of Health instituted, in 2018, a project to identify the main etiological agents of genital ulcers (HSV type 1 and 2, *T. pallidum*, and *H. ducreyi*) using molecular tests in specific services. This project's findings may modify the current conduct flowchart regarding genital ulcers, as was the case for urethral discharge syndrome^32^ ^,^ ^33^.

During the evaluation of genital ulcer complaints, given evidence or history of characteristic vesicular lesions, the treatment of genital herpes is indicated. In case of first episode, acyclovir 200mg, two pills, *per os*, three times a day, for seven to ten days, and, in the case of recurrence, acyclovir 200mg, two tablets, *per os*, three times a day, for five days. In cases of need of genital herpes suppression (six or more episodes per year), acyclovir 200mg, two pills, *per os*, twice a day, for up to six months, with the possibility of prolonging the treatment for up to two years. 

**FIGURE 2:**
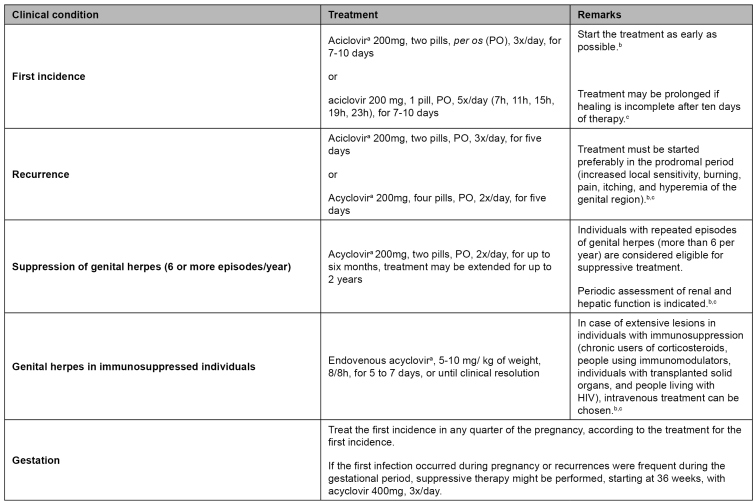
Treatment of genital herpes.

**Source:** Clinical Protocol and Therapeutic Guidelines for Comprehensive Care for People with Sexually Transmitted Infections, 2020^1^.

**Notes:** a) Treatment with antivirals is effective in reducing the intensity and duration of incidences when performed early; b) Local treatment can be done with compresses of physiological solution or a degerming aqueous solution to sanitize the lesions. Oral analgesics can be used if necessary. It is recommended to return within one week to reevaluate the lesions; c) The means of transmission, the possibility of asymptomatic infection, the fear of rejection by sexual partners, and concerns about the ability to have children are aspects that must be addressed. It is important to mention that there is no association between genital herpes and cancer.

**FIGURE 3:**
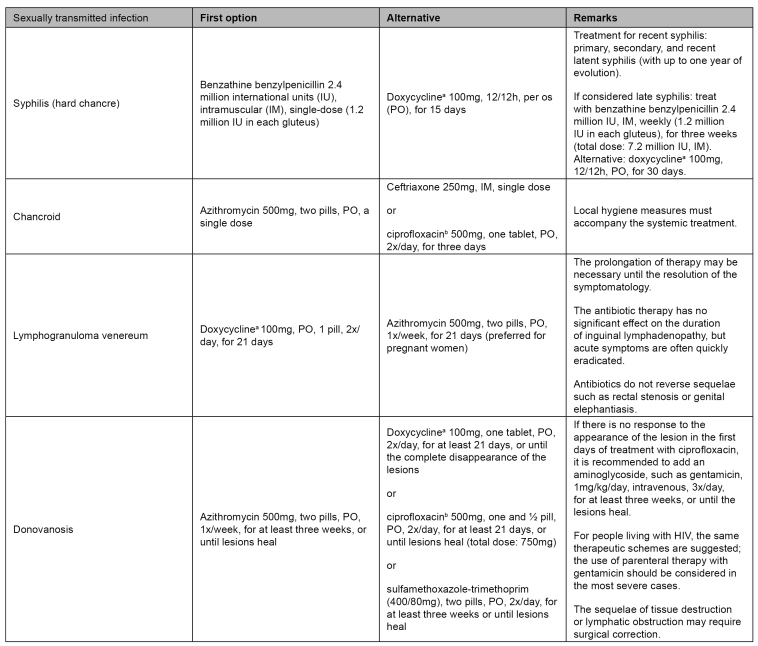
Treatment of genital ulcers with a diagnosis of syphilis, chancroid, lymphogranuloma venereum, or donovanosis.

**Source:** adapted from the Clinical Protocol and Therapeutic Guidelines for Comprehensive Care of People with Sexually Transmitted Infections, 2020^1^ and CDC guidelines, 2015^9^.

**Notes:** a) Doxycycline is contraindicated for pregnant and lactating women; b) Ciprofloxacin is contraindicated for children and pregnant and lactating women.

For the treatment of other STI with genital ulcers and clinical pictures with less than four weeks of evolution, syphilis (hard chancre) must be treated with benzathine penicillin, a single dose of 2.4 million international units (IU), deep intramuscular (1.2 million IU in each gluteus), and chancroid with azithromycin 1g, single-dose, *per os*. In cases of lesions with more than four weeks of evolution, a biopsy must be performed. The institution of treatment for syphilis, chancroid, lymphogranuloma venereum, and donovanosis must be assessed. For lymphogranuloma venereum and donovanosis, more prolonged treatments are necessary. In the case of lymphogranuloma, doxycycline 100mg must be taken twice a day orally for 21 days as the first option, and azithromycin as an alternative. In the case of donovanosis, it is recommended to take azithromycin 500mg, two pills, *per os*, once a week, for at least three weeks or until the lesions heal, and, as an alternative treatment, the use of doxycycline, ciprofloxacin, or sulfamethoxazole-trimethoprim may be assessed^1^. The recommended treatments, including in special situations such as immunosuppression and pregnancy, are summarized in Figure 2 and Figure 3. It is important to emphasize that given a lesion with sudden evolution and a history of exposure to drugs, fixed drug eruption must be considered^24^ ^,^ ^34^. 

The sexual partners of the last three months must be oriented about exposure risks and evaluated and treated according to clinical, laboratory, or epidemiological findings^8^ ^,^ ^9^ ^,^ ^12^ ^,^ ^24^ ^,^ ^25^. Sexual partners of individuals with syphilis whose exposure occurred up to 90 days before the onset of symptoms must be evaluated^19^ ^,^ ^20^, performing their presumptive treatment with a single dose penicillin benzathine 2.4 million IU, single-dose, intramuscular. Sexual partners of individuals with chancroid whose exposure occurred up to ten days before the onset of symptoms should be evaluated and treated with azithromycin 500mg, two pills, *per os*, single dose^9^ ^,^ ^12^. For asymptomatic partners of people with HSV-1 and HSV-2, presumptive treatment is not recommended^8^ ^,^ ^31^ ^,^ ^35^. Presumptive treatment of sexual partners of people with chlamydia, with exposure up to 60 days before the onset of symptoms is indicated^9^ ^,^ ^14^ ^,^ ^22^ ^,^ ^28^ with azithromycin 500mg, two pills, *per os*, single-dose, or doxycycline 100mg, one tablet, *per os*, twice a day for seven days (contraindicated in pregnant women, nursing mothers and children under nine). In the case of donovanosis, presumptive treatment of asymptomatic sexual partners is not recommended because of low infectivity^16^ ^,^ ^29^.

### Surveillance, prevention, and control

Counseling centered on the person and their sexual practices aim at recognizing riskier practices and establishing a risk reduction plan in light of combined prevention recommendations for STI, HIV, and viral hepatitis^1^. The use of barrier methods during oral, vaginal, and anal sexual activity must be indicated. Sexual accessories must be for individual use and sanitized before, and after use^1^ ^,^ ^9^. HIV infection prophylaxis on post-exposure or pre-exposure must be offered when indicated^1^. 

Rapid testing for HIV, syphilis, and hepatitis B and C infection must invariably be recommended. Post-therapeutic serological follow-up tests are indicated for people with syphilis. Vaccination for HPV and hepatitis A and B prophylaxis should follow the recommendations^9^ ^,^ ^24^ ^,^ ^25^. NAAT screening for *C. trachomatis* and *Neisseria gonorrhoeae* infections in urine samples, urethral or cervical exudate is recommended, despite its unavailability in most services^1^.

Syphilis cases with ulcers that present a reagent test (treponemal or nontreponemal) must be compulsorily reported as acquired syphilis^31^, and partners will only be notified after investigation, depending on the test results and the presence of symptoms, as defined in the case definition of acquired syphilis^1^. 

## SPECIAL POPULATIONS

### Pregnant women

Genital ulcers caused by STI deserve careful attention due to the potential for vertical transmission of some etiological agents, especially *T. pallidum*. The suspicion of syphilis in pregnant woman indicates treatment according to the clinical stage, in a mandatory way, independently of laboratory results. Post-treatment nontreponemal tests must be performed every 30 days^9^ ^,^ ^19^ ^,^ ^20^. HSV also presents specificities in its management during pregnancy. Its occurrence at the end of pregnancy increases the risk of fetal and neonatal complications. In the presence of active lesions in the genital area, a cesarean section is indicated^8^ ^,^ ^36^ ^,^ ^37^. The occurrence of adverse events in pregnancy due to chancroid and donovanosis is rare^9^. In the case of donovanosis, there are reports of perinatal transmission cases involving the otorhinolaryngological structures of the newborn^38^ ^-^ ^40^.

### People with HIV infection

STI treatment regimens are the same as those recommended for people without HIV infection. Genital ulcers facilitate HIV transmission, increasing the importance of early treatment, and the higher risk is especially significant for genital herpes, syphilis, and chancroid^9^ ^,^ ^23^ ^,^ ^24^. Syphilis coinfection can alter the clinical course of this condition, with atypical and aggressive manifestations^19^ ^,^ ^20^. In HSV coinfection, lesions can be more painful, atypical, and longer-lasting and may require intravenous medication to control the symptoms^8^ ^,^ ^35^. Chancroid and lymphogranuloma venereum must be carefully monitored since they may need more prolonged treatment due to greater chance of response delay or therapeutic failure^14^ ^,^ ^21^ ^,^ ^27^.
